# Importance of multimodality imaging in the differential diagnosis of an intra-atrial septum mass: a case report

**DOI:** 10.1093/ehjcr/ytag236

**Published:** 2026-03-20

**Authors:** Isabel Martins Moreira, Marta Catarina Bernardo, Inês Silveira, Ilídio Moreira

**Affiliations:** Department of Cardiology, Hospital Centre of Tras-os-Montes and Alto Douro, Avenida da Noruega, Vila Real 5000-508, Portugal; Department of Cardiology, Hospital Centre of Tras-os-Montes and Alto Douro, Avenida da Noruega, Vila Real 5000-508, Portugal; Department of Cardiology, Hospital Centre of Tras-os-Montes and Alto Douro, Avenida da Noruega, Vila Real 5000-508, Portugal; Department of Cardiology, Hospital Centre of Tras-os-Montes and Alto Douro, Avenida da Noruega, Vila Real 5000-508, Portugal

## Case description

**Figure 1:**
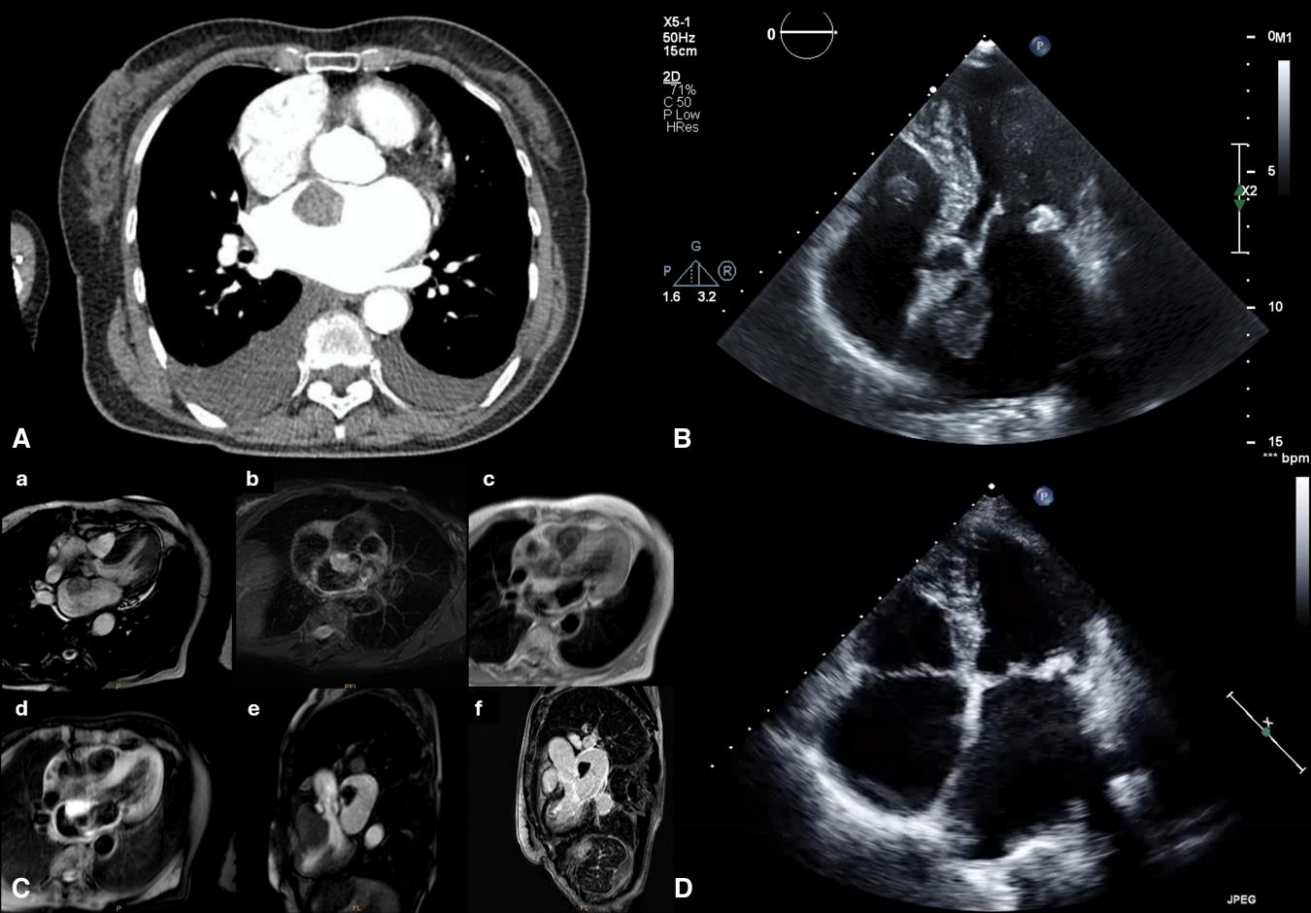
(*A*) Computed tomography (CT) pulmonary angiography demonstrating a large mass in the left atrium; (*B*) Transthoracic echocardiography (5-chamber view) revealing a mobile, heterogeneous mass (30 × 20 mm), attached to the interatrial septum. This image corresponds to Supplementary material online, Videos S1-S2; (*C*) Cardiac magnetic resonance: (C-a) Cine image SSFP sequence of the left atrial mass, (C-b) High-intensity signal on the fluid-sensitive sequence (T2 STIR), (C-c) High-intensity signal on the fluid-sensitive sequence (T1 STIR), (C-d) No signal on the fat-suppressed image; (C-e) No first-pass perfusion, (C-f) No contrast enhancement. This image corresponds to Supplementary material online, *Figure S2*; (*D*) Follow-up transthoracic echocardiography demonstrating complete resolution of the thrombus. SSFP: steady-state free precession, STIR: short time inversion recovery.

An 82-year-old woman with arterial hypertension and left bundle branch block presented to the emergency department with progressive exertional dyspnea, orthopnea, and palpitations. At admission, the patient was tachypneic and tachycardic, with bibasilar inspiratory crackles on pulmonary auscultation. Electrocardiogram revealed *de novo* atrial fibrillation with rapid ventricular response (see Supplementary material online, *Figure S1*), and laboratory tests showed elevated NT-proBNP and D-dimer levels. Computed tomography pulmonary angiography excluded pulmonary embolism but incidentally identified a large left atrial mass (*Figure 1A*). Transthoracic echocardiography demonstrated severe posterior mitral annulus calcification with mild mitral stenosis, severe left atrium dilatation, preserved biventricular systolic function and a mobile heterogeneous mass measuring 30 × 20 mm, attached to the interatrial septum (*Figure 1B* and Supplementary material online, *Videos S1*–*SS2*).

The patient was hospitalized for monitoring and further evaluation. Cardiac magnetic resonance (CMR) was performed to better characterize the mass, revealing an oval structure, hyperintense on T1- and T2-weighted images, without perfusion, fat-saturation signal, or gadolinium enhancement—findings suggestive of a recent thrombus (*Figure 1C*). She was discharged on warfarin, bisoprolol and digoxin, and follow-up echocardiography at 3 months demonstrated complete resolution of the thrombus (*Figure 1D*).

The diagnostic workup of a cardiac mass should integrate knowledge of tumour type, epidemiology and imaging characteristics.^1^ Multimodality imaging is essential for the non-invasive assessment of cardiac masses.^2^ The left atrium is the most frequent location for both thrombi and myxomas. On CMR, thrombi are typically hypointense on all sequences, whereas myxomas exhibit homogeneous hyperintensity on T2- and isointensity on T1-weighted images. Recent thrombi may mimic myxomas by appearing hypertense on both sequences. In such cases, the absence of gadolinium enhancement becomes a key discriminator, favouring thrombus over tumour.^3^ This case highlights the pivotal role of CMR tissue characterisation, particularly the assessment of gadolinium enhancement, in distinguishing atrial thrombi from cardiac tumours.

## Supplementary Material

ytag236_Supplementary_Data

## Data Availability

No new data was generated or analysed in support of this research.
