# Supplementary material for: Importance of multimodality imaging in the differential diagnosis of an intra-atrial septum mass: a case report
Source: Eur Heart J Case Rep. 2026 Mar 20;10(4):ytag236. doi: 10.1093/ehjcr/ytag236 (PMC13042243; doi:10.1093/ehjcr/ytag236)
Supplement: ytag236_Supplementary_Data [file ytag236_supplementary_data.zip › Supplementary Table S1.docx]

**Supplementary Table S1. Imaging features differentiating atrial thrombi and atrial myxomas**

| **Feature** | **Atrial thrombus** | **Atrial myxoma** |
| --- | --- | --- |
| Typical clinical context | Atrial fibrillation, low-flow states, mitral valve disease | Sporadic; may be associated with constitutional symptoms |
| Typical location | Left atrial appendage or posterior wall | Interatrial septum, typically at the fossa ovalis |
| Attachment | Broad-based or mural | Pedunculated, narrow stalk |
| Mobility | Variable | Usually mobile |
| T1-weighted CMR | Hypointense (chronic); hyperintense if recent | Isointense |
| T2-weighted CMR | Hypointense (chronic); hyperintense if recent | Hyperintense |
| First-pass perfusion | Absent | Present |
| Late gadolinium enhancement | Absent | Present |
| Response to anticoagulation | Progressive reduction or resolution | No change |
