# Supplementary figures and images for: Importance of multimodality imaging in the differential diagnosis of an intra-atrial septum mass: a case report
Source: Eur Heart J Case Rep. 2026 Mar 20;10(4):ytag236. doi: 10.1093/ehjcr/ytag236 (PMC13042243; doi:10.1093/ehjcr/ytag236)

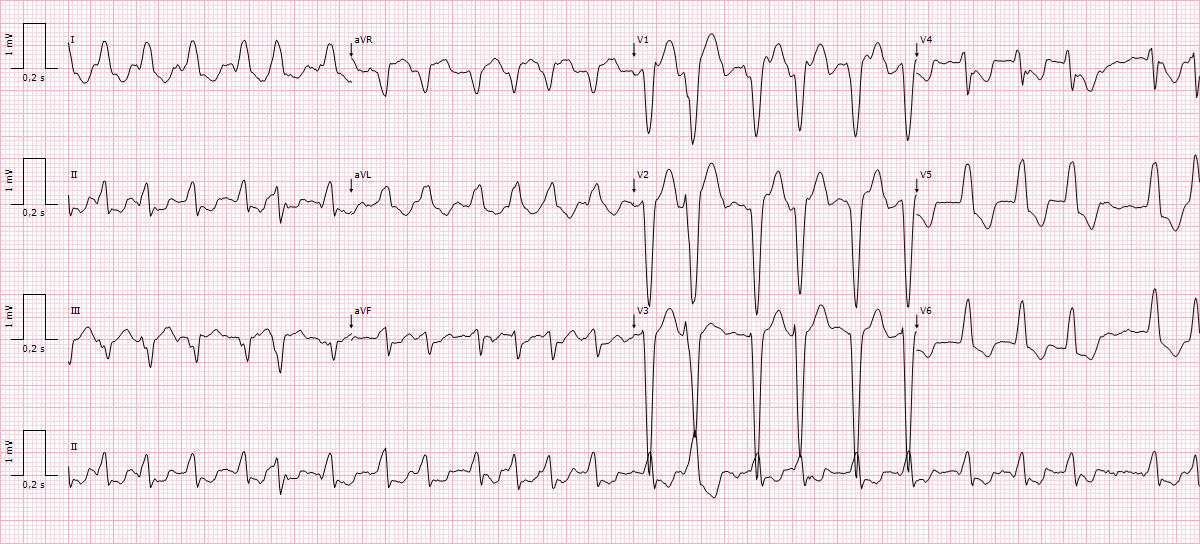

Supplement: ytag236_Supplementary_Data [file ytag236_supplementary_data.zip › Figure S1.png]

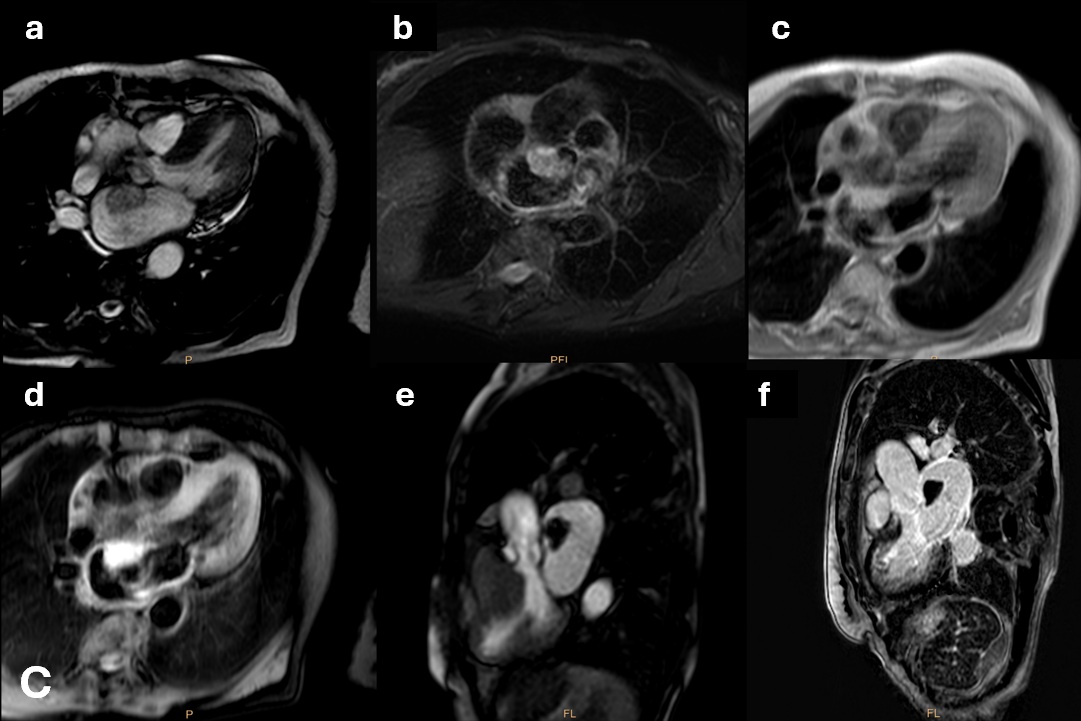

Supplement: ytag236_Supplementary_Data [file ytag236_supplementary_data.zip › Figure S2.jpg]
